# Fractions and Compounds Obtained from Transformed Plant Cell Cultures of *Lopezia racemosa* Show Anti-Inflammatory and Cytotoxic Activities

**DOI:** 10.3390/plants14162585

**Published:** 2025-08-20

**Authors:** Lizbeth Coronel-Pastor, María Luisa Villarreal, Alejandro Zamilpa, Maribel Herrera-Ruiz, Manases González-Cortazar, Laura Alvarez, Irene Perea-Arango, Norma Elizabeth Moreno-Anzúrez, Mario Rodríguez Monroy, José de Jesús Arellano-García

**Affiliations:** 1 Centro de Investigación en Biotecnología, Universidad Autónoma del Estado de Morelos, Av. Universidad 1001, Cuernavaca CP 62209, Morelos, Mexico; lizbeth.coronel@uaem.edu.mx (L.C.-P.); luisav@uaem.mx (M.L.V.); iperea@uaem.mx (I.P.-A.); 2 Centro de Investigación Biomédica del Sur, Instituto Mexicano del Seguro Social, Argentina No. 1, Xochitepec CP 62790, Morelos, Mexicogmanases@hotmail.com (M.G.-C.); 3 Centro de Investigaciones Químicas, Universidad Autónoma del Estado de Morelos, Av. Universidad 1001, Cuernavaca CP 62209, Morelos, Mexico; lalvarez@uaem.mx; 4 Centro de Desarrollo de Productos Bióticos, Instituto Politécnico Nacional, Carretera Yautepec-Jojutla Km 6, Calle CEPROBI No. 6 Colonia San Isidro, Yautepec CP 62731, Morelos, Mexico; nmorenoa@ipn.mx (N.E.M.-A.); mrmonroy@ipn.mx (M.R.M.)

**Keywords:** cytotoxic activity, hairy roots, anti-inflammatory activity, maslinic acid, corosolic acid

## Abstract

*Lopezia racemosa* Cav., commonly known as “cancer herb” in indigenous communities, has long been used for its medicinal properties. The biotechnological production of its bioactive compounds through genetic transformation represents a valuable approach for obtaining pharmacologically relevant substances. The initial focus of this study was to identify compounds previously reported in the species; however, phytochemical analysis by HPLC and NMR led to the isolation and identification of two pentacyclic triterpene esters not previously described in *L. racemosa*: 3-O-[(E)-feruloyl]-maslinic acid (**1**) and 3-O-[(E)-feruloyl]-corosolic acid (**2**), identified as constituents of fraction 33. The LRTC3.1 callus line was obtained from hairy roots generated by infecting *L. racemosa* leaf explants with *Agrobacterium rhizogenes* strain ATCC15834/pTDT. The crude extract, specific fractions, and the mixture of these compounds demonstrated significant anti-inflammatory and cytotoxic activities. Anti-inflammatory activity was evaluated using the carrageenan-induced mouse paw edema model, where the crude extract achieved 51.02% inhibition of inflammation compared to meloxicam (30.86%). Cytotoxicity was assessed against three human cancer cell lines: breast carcinoma (MCF7), cervical carcinoma (SiHa), and colon carcinoma (HCT-15). Fractions FD (28–29) and 33 exhibited potent cytotoxic effects, with IC_50_ values of 0.508 and 1.345 µg/mL against SiHa cells, and 0.053 and 2.693 µg/mL against MCF-7 cells, respectively. These findings suggest that transformed *L. racemosa* cultures represent a promising source of bioactive compounds for potential therapeutic development.

## 1. Introduction

*Lopezia racemosa*, a species traditionally used in Mexican medicine, represents a promising source of pharmacologically active compounds. This plant, commonly found in crop plantations or along roadsides, is considered a wild undergrowth species and is widely distributed across the American continent, from northern Mexico to El Salvador. Depending on environmental conditions, *L. racemosa* may exhibit annual or perennial growth, typically flowering between September and November [1,2,3,4]. In the Mexican Mixtec communities, it is known as Yua xnteé, while other common names include Perita, Guayabilla, Punch herb, and cancer herb. This plant is primarily used for medicinal purposes, though it can also be edible and mixed with corn straw as cattle feed [5,6]. According to ethnobotanical sources, including the Atlas of the Plants of Mexican Traditional Medicine [7], *L. racemosa* is traditionally employed to treat skin infections, inflammation, gastrointestinal ailments, and fever. The aerial parts are commonly prepared as infusions or decoctions, applied topically to wounds and rashes, or ingested to alleviate internal conditions. Its traditional uses also include relief from menstrual pain and gastrointestinal cramps, highlighting its ethnopharmacological relevance.

Previous studies have reported several pharmacological activities in organic extracts of this species, including antibacterial, anti-inflammatory, antiparasitic, antifungal, and cytotoxic properties [8]. Our research group has previously reported findings on *L. racemosa*, including the culturing of callus cultures by adding plant growth regulators to the culture medium and the isolation of two sterol derivatives with anti-inflammatory activity, 6-O-palmitoyl-3-O-β-D-glucopyranosylcampesterol and 6-O-palmitoyl-3-O-β-D-glucpy ranosyl-β-sitosterol [9]. We have also reported the production of two terpene acids, ursolic and oleanolic, and a phytosterol derivative (campesterol) from of CH_2_Cl_2_, methanol (1:1), which was the major component of the active fraction and showed cytotoxic and anti-inflammatory activities in a hairy root culture, (23R)-2α,3β,23,28-tetrahydroxy-14,15-dehydrocampesterol (3), obtained from the LRT7.31 cell line [10]. These findings led to the study of the LRT3.1 cell line, a *L. racemosa* line previously established in the laboratory as part of the genetic transformation protocol mediated by *A. rhizogenes* (ATCC 15834/pTDT), developed by Moreno-Anzures [10]. This line was selected because neither the biological effect nor the active compounds were known, and it was projected that it would produce the same chemical compounds (terpenoids and phytosterols) previously described, with the aim of preserving the anti-inflammatory and cytotoxic effects of the extract and its fractions. Therefore, our approach focused on the search, isolation, and identification of these compounds from the extract.

## 2. Results

### 2.1. Fractionation

**Scheme 1:**
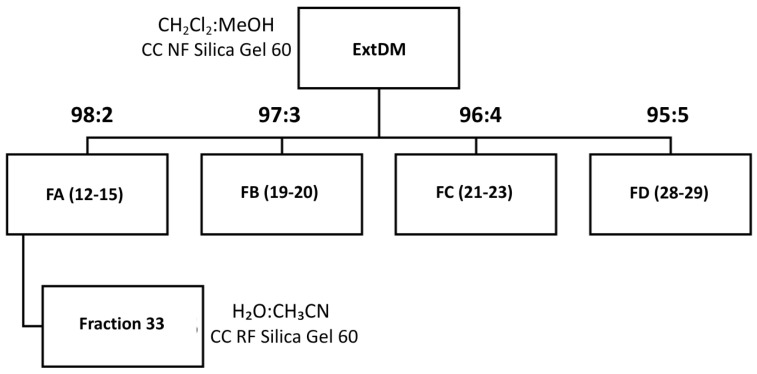
Chemical fractioning. Summary of obtaining the fractions and isolation of compounds **1** and **2** from the crude extract. The fractions were analyzed by means of normal-phase and reverse-phase thin-layer chromatography.

Scheme 1 shows the fractionation procedure carried out during the phytochemical analysis. A detailed description of the fractioning procedure is in the Section 4.

### 2.2. Detection of rol Genes and Verification of Genetic Transformation in LRTC3.1

**Figure 1:**
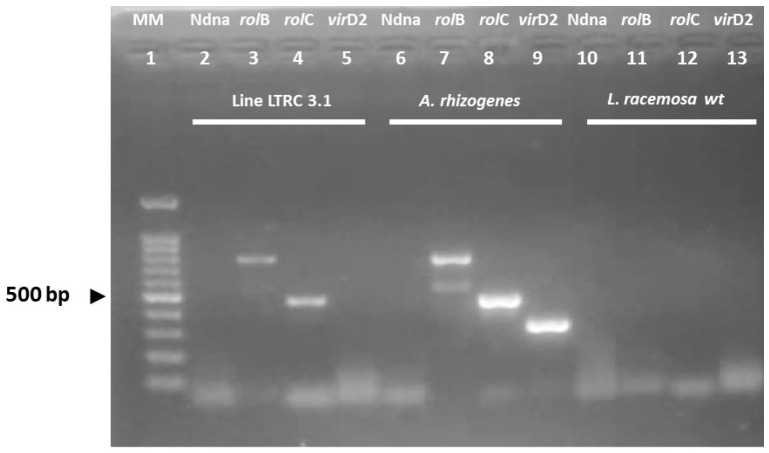
PCR analysis. PCR products from DNA of the transformed cell line LRTC3.1; DNA from *A. rhizogenes* strain ATCC15834/pTDT and DNA of the wild-type *L. racemosa* plant. Lane 1, molecular marker (MM); PCR control lanes 2, 6, and 10 no amplification of the PCR mixture without DNA (Ndna); lanes 3 and 4, *rol*B and *rol*C amplified gene fragments in LRT3.1 (652 and 490 bp, respectively); lane 5, no amplification of *vir*D2 in LRTC3.1; lanes 7, 8, and 9 amplified gene fragments of *rol*B, *rol*C, and *vir*D2 gene fragments in *A. rhizogenes* ATCC15834/pTDT (652, 490, and 338 bp, respectively); lanes 11 to 13, no amplification in any of the *rol* genes nor *vir*D2 in the negative control *L. racemose* wild-type DNA.

PCR analysis was performed on genomic DNA from the LRTC3.1 cell line, using *A. rhizogenes* ATCC15834/pTDT DNA as a positive control and wild-type *L. racemosa* DNA as a negative control. LRTC3.1 yielded the expected 652 bp *rol*B and 490 bp *rol*C fragments, with no amplification of *vir*D2, confirming transgene integration and the absence of bacterial contamination. In contrast, the positive control amplified *rol*B, *rol*C, and a 338 bp *vir*D2 fragment, while the wild-type plant DNA showed no amplification of any *rol* or *vir*D2 genes (Figure 1).

### 2.3. Compounds Elucidation by NMR and ESI-MS

**Figure A1:**
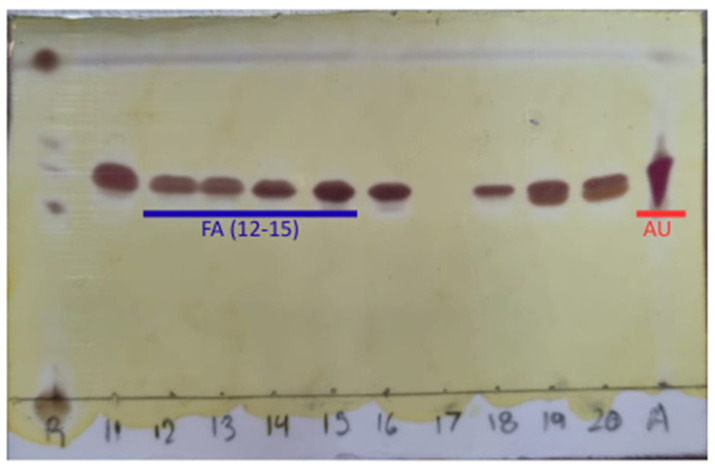
Chromatographic fractionation of ExtDM yielded fraction FA (12–15); reference ursolic acid (AU).

**Figure A2:**
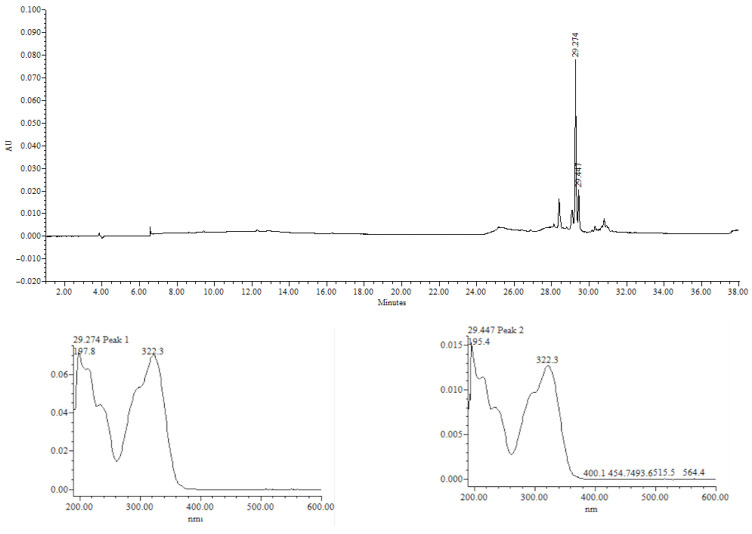
HPLC chromatogram and UV spectrum. Detection for isolated compounds **1** and **2** was performed at 325 nm.

Chromatographic fractionation of ExtDM yielded fraction FA (12–15), in which, when compared by thin-layer chromatography (TLC) with terpene standards (ursolic acid; see Appendix A; Figure A1), the presence of the target active compounds was identified, thus allowing the isolation of compounds **1** and **2**. Subsequent high-performance liquid chromatography (HPLC) analysis of the isolated compounds revealed a retention time of 29.27 min and a UV-Vis spectrum λmax of 197.8 and 322.3 nm (**1**); and a retention time of 29.44 min, and a UV-Vis spectrum λmax of 195.4 and 322.3 nm (**2**), features consistent with the phenylpropanoid (see Appendix A; Figure A2).

Compounds **1** and **2** were isolated from the FA (12–15) fraction as a white, amorphous powder through multiple chromatographic separations. The yield was 12 mg.

**Figure 2:**
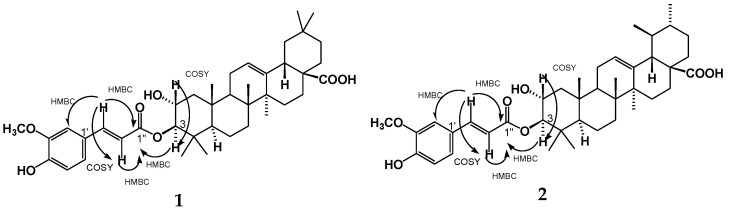
HMBC and COSY correlations of compounds **1** and **2**.

**Figure A3:**
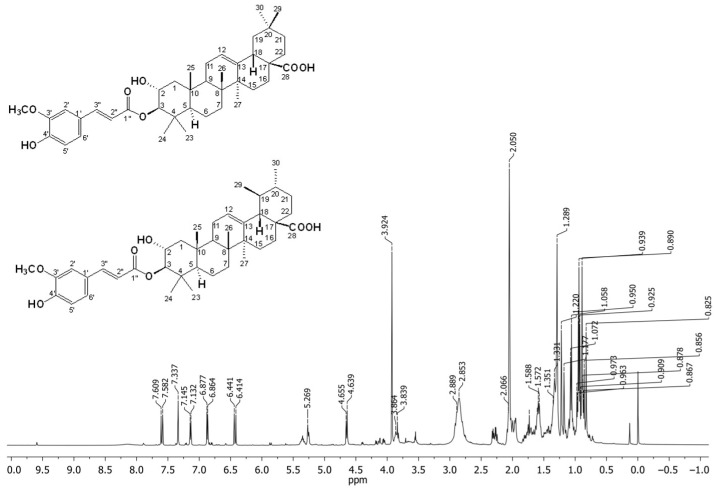
^1^H NMR (CDCl_3_, 600 MHz) of compounds **1** and **2**.

**Figure A4:**
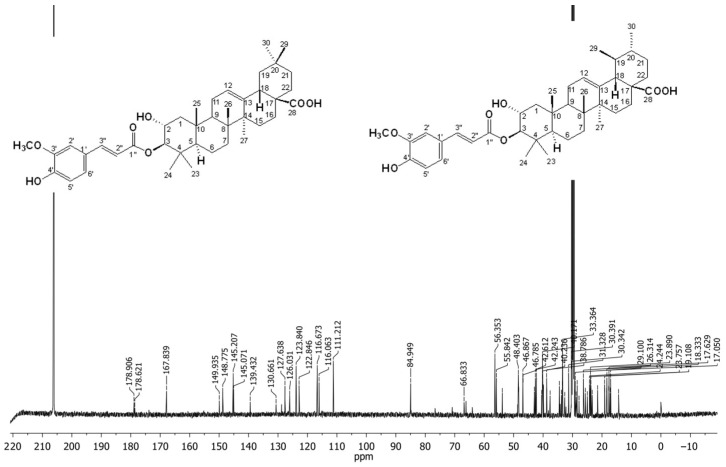
^13^C NMR (CDCl_3_, 150 MHz) of compounds **1** and **2**.

**Figure A5:**
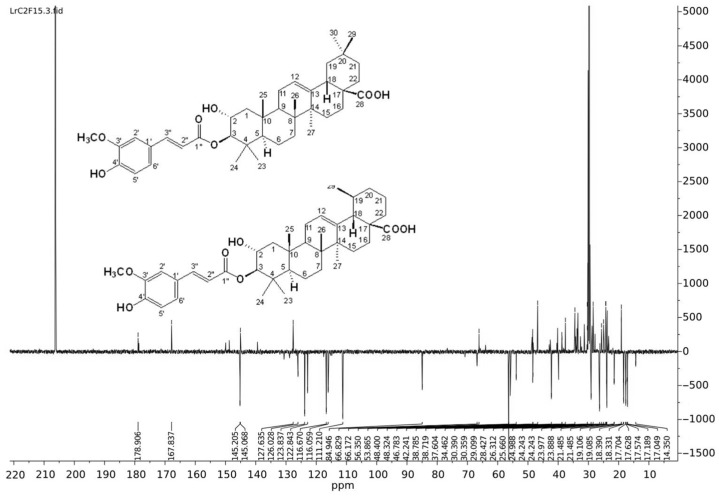
^13^C (DEPT) NMR (CDCl_3_, 600 MHz) of compounds **1** and **2**.

**Figure A6:**
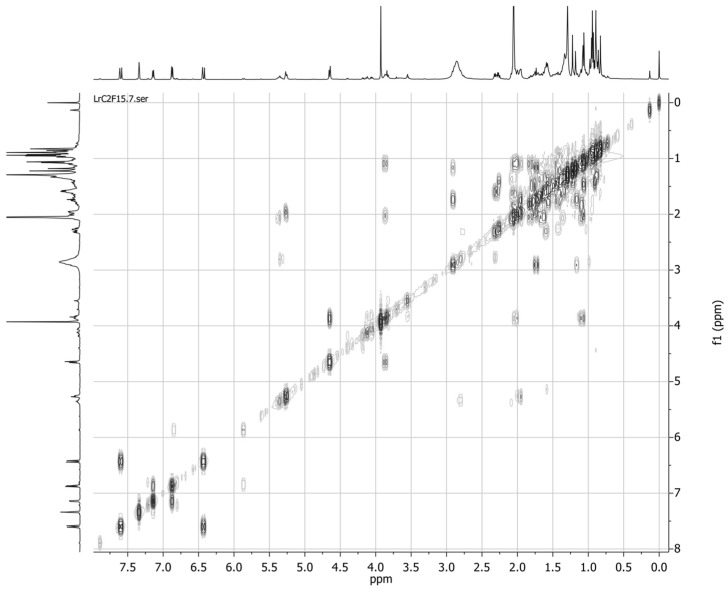
^1^H-^1^H COSY NMR (CDCl_3_, 600 MHz) of compounds **1** and **2**.

**Figure A7:**
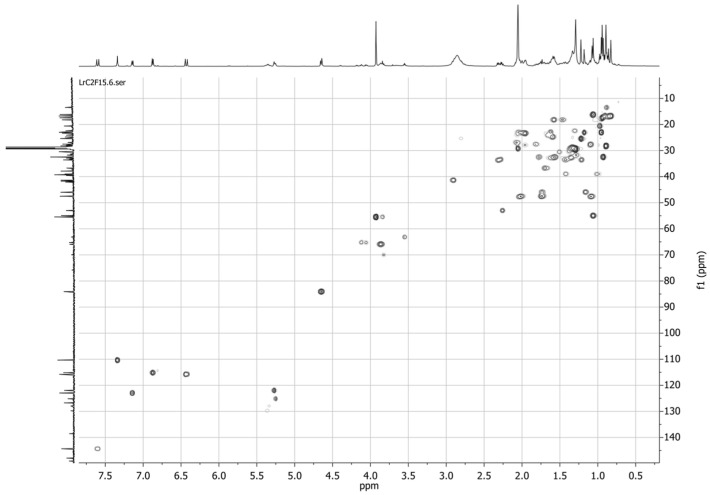
^1^H-^13^C (HSQC) NMR (CDCl_3_, 600 MHz) of compounds **1** and **2**.

**Figure A8:**
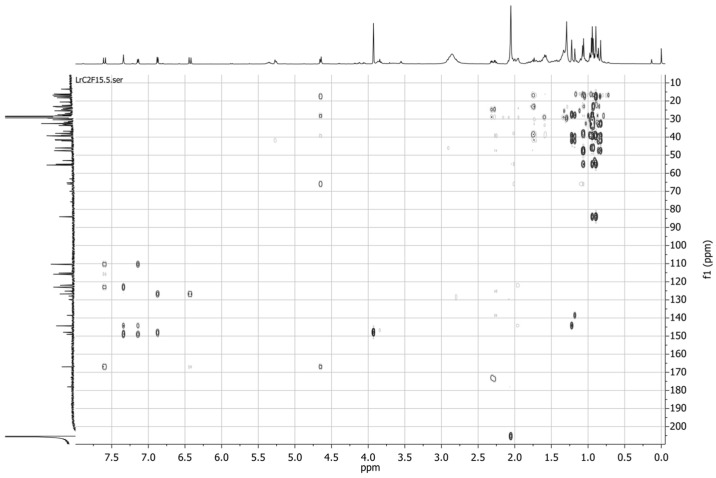
^1^H-^13^C (HMBC) NMR (CDCl_3_, 600 MHz) of compounds **1** and **2**.

The ^1^H-NMR spectrum of **1** exhibited an ABX aromatic ring system at δ 7.33 (s, br, H-2′), 6.87 (d, 8.1 Hz, H-5′), 7.14 (d, br, 8.1 Hz, H-6′) and a double bond system at δ 6.42 (d, 15.8, H-2″) and 7.59 (d, 15.8 Hz, H-3″). The coupling constant of H-2″ and H-3″ indicated the trans position. The analysis of the spectroscopic data of this part of the molecule corresponds to ferulic acid. In the high-field ^1^H-NMR analysis, seven singlets at δ 0.82 (s, H-26), 0.89 (s, H-23), 0.94 (s, H-24), 1.05 (s, H-25), 1.22 (s, H-27, H-30) and 1.28 (s, H-29) suggest a triterpene. The DEPT experiment corroborated the presence of seven methyls, nine methylenes, six methines, and eight quaternaries through carbon analysis. The HMBC experiment of H-2″ and H-3″ correlated with the carbonyl at δ 167.8 assigned to H-1″ of ferulic acid. H-1′′ (C=O) correlates to three ligations (HMBC) with a doublet signal of an oxygenated base at δ 4.63 (d, 9.7 Hz), assigned to H-3 in the alpha position of the triterpene skeleton, thus confirming the union of ferulic acid with triterpene (Figure 2) (see Appendix A; Figure A3, Figure A4, Figure A5, Figure A6, Figure A7 and Figure A8). Furthermore, H-3 shows a correlation in the COSY experiment with a methine-type oxygen base multiplet signal (CH) at δ 3.86 (m) and is assigned to H-2 (OH in α position). In the negative-ion ESI-MS of **1**, quasimolecular ion peaks at *m*/*z* 647.36 [M-H]^−^, and 455.5 [M-C_10_H_9_O_4_]^−^ were observed. Therefore, according to the analysis of the spectroscopic data of this triterpene and those described in the literature, they correspond to the mixture of the pentacyclic triterpenes known as maslinic and corosolic acids [11] (see Appendix A; Table A1).

**Figure 3:**
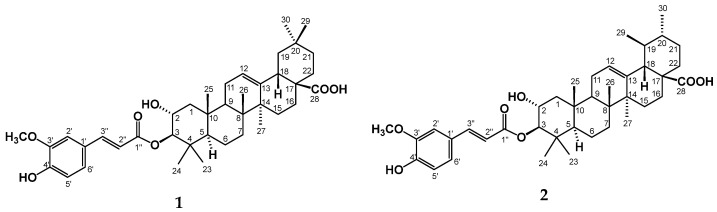
3-O-[(E)-feruloyl]-maslinic (**1**) and 3-O-[(E)-feruloyl]-corosolic acid (**2**).

Therefore, these molecules are called 3-O-[(E)-feruloyl]-maslinic acid (**1**) and 3-O-[(E)-feruloyl]-corosolic acid (**2**) (Figure 3) [12,13]. These compounds have not been previously reported in this species. Although they were not the expected compounds that had been reported for the other cell line, we considered it important to test their anti-inflammatory and cytotoxic activity.

### 2.4. Evaluation of the Anti-Inflammatory Activity by the Carrageenan Assay

**Figure 4:**
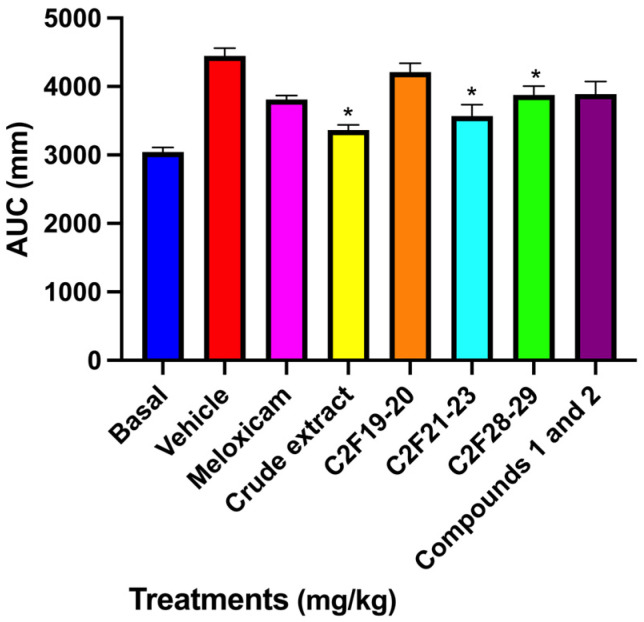
AUC of the curve of edema/thickness (mm) in the mouse paw. Data is shown as media ± SE (*n* = 7), with one-way ANOVA, and the statistical difference is * *p* ≤ 0.05 when compared with the vehicle group.

The mixture of compounds **1** and **2** was evaluated in the anti-inflammatory model and showed an effect; however, it was not statistically significant (Table 1 and Table 2, Figure 4). Since this mixture, obtained from fraction FA (12–15), did not exhibit a relevant effect, the remaining fractions were subsequently tested to determine where the biological activity was concentrated.

Results are summarized in Table 1. In the meloxicam-treated group (positive control), paw edema increased continuously but remained significantly lower than in the vehicle group (control), reaching 2.71 ± 0.10 mm versus 3.38 ± 0.19 mm at 4 h. Likewise, the ExtDM and fractions FC (21–23) and FD (28–29) produced a statistically significant anti-inflammatory effect compared to the vehicle, with maximum edema values of 2.68 ± 0.26 mm (*p* < 0.0003), 2.65 ± 0.32 mm (*p* < 0.0003), and 2.85 ± 0.34 mm (*p* < 0.012), respectively, at the 4 h time point.

The time-course data (0–24 h) were further summarized by calculating the area under the edema-time curve (AUC) for each treatment using the standard trapezoidal rule, which provides a single metric of overall anti-inflammatory efficacy. These AUC values (Table 2) confirm and extend the time point analysis: as shown in Figure 1, the ExtDM and fractions FC (21–23) and FD (28–29) produced a significant reduction in AUC compared to the vehicle control indicating an anti-inflammatory effect.

The highest inflammation value was reached at 4 h in all treatments except for meloxicam and FB (19–20). A more notable reduction in inflammation was observed in the ExtDM (51.02%) and FC (21–23) fraction (27.98%), followed by the FB (19–20) fraction (23.45%), FD (28–29) fraction (22.22%), and the fraction 33 (compounds **1** and **2**) (18.51%). In contrast, the meloxicam treatment resulted in a 30.86% decrease in inflammation (Table 2).

### 2.5. Cytokine Analysis

**Figure 5:**
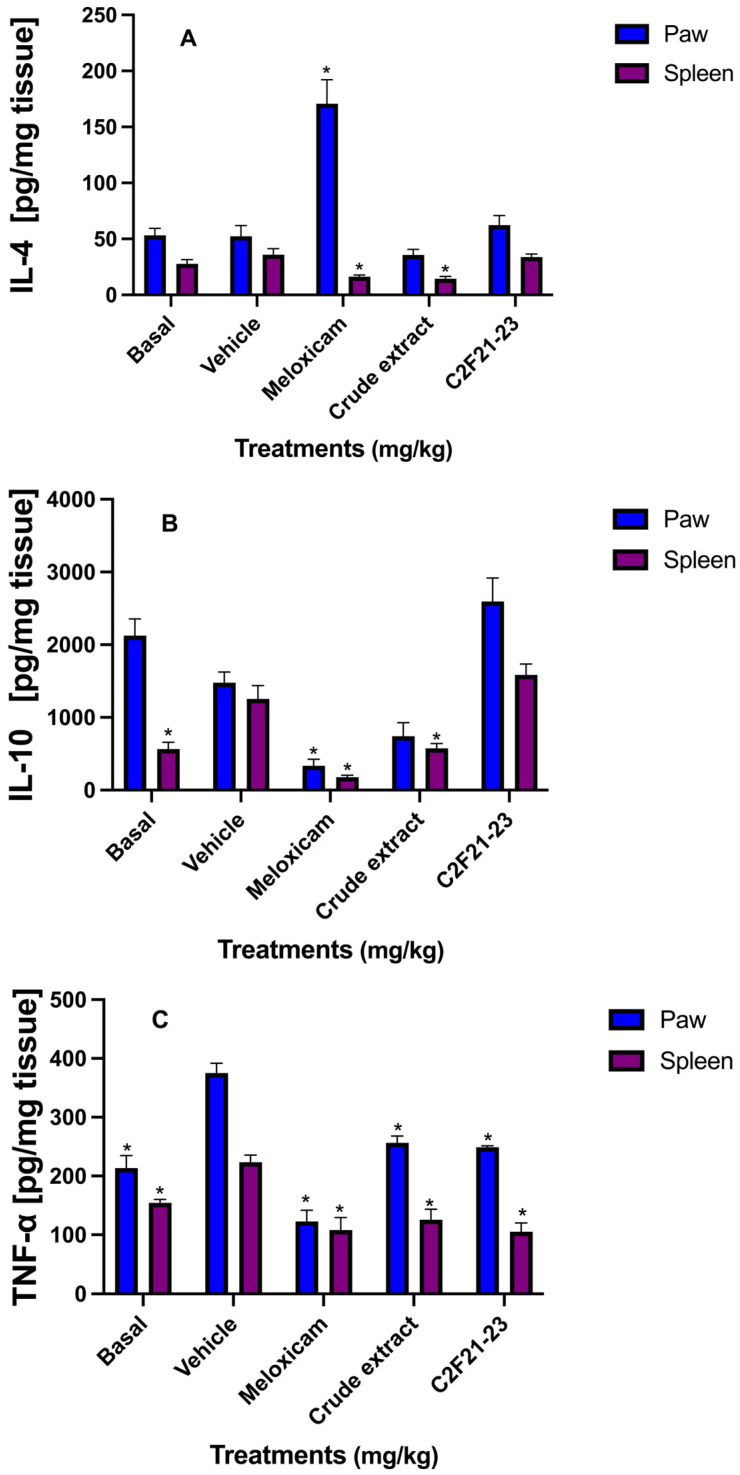
Effect of the ExtDM and the FC (21–23) fraction on the la concentration of IL-4 (**A**), IL-10 (**B**), and TNF-α (**C**) regarding the vehicle control. Data are presented as mean ± SE (*n* = 5). Statistical significance was determined by one-way ANOVA followed by Dunnett’s post hoc test compared treatment with the vehicle (* *p* ≤ 0.05).

The concentrations of cytokines in the spleen and paw were evaluated. The highest values of IL-4 were found in the meloxicam/paw group (170.76 ± 47.72 pg/mg; *p* < 0.002) relative to the vehicle (Figure 5A). In contrast, IL-10 was increased in the group treated with the fraction FC (21–23)/paw, at 2595.35 ± 647.84 pg/mg of tissue (Figure 5B). In the case of TNF-α, a decrease was primarily observed in the meloxicam group (spleen 108.18 ± 46.93 pg/mg; paw 122.66 ± 43.25 pg/mg), the ExtDM (spleen 125.80 ± 35.69 pg/mg; *p* < 0.001; paw 256.40 ± 26.22 pg/mg; *p* < 0.029), and the FC (21–23) fraction (spleen 105.06 ± 30.36 pg/mg; paw 249.04 ± 5.1 pg/mg; *p* < 0.018). This finding was in comparison to the vehicle (spleen 223.58 ± 26.77 pg/mg; paw 351.90 ± 61.60 pg/mg) (Figure 5C).

### 2.6. Evaluation of Cytotoxic Activity

The cytotoxic activity of the ExtDM, along with the FD (28–29) and fraction 33 (compounds **1** and **2**) was evaluated against three human cancer cell lines: breast carcinoma (MCF7), cervical carcinoma (SiHa), and colon carcinoma (HCT-15) (Table 3). For the ExtDM, the obtained IC_50_ values were 7.777 ± 0.534 µg/mL for SiHa, 4.837 ± 0.129 µg/mL for HCT-15, and 2.507 ± 0.121 µg/mL for MCF7. The FD (28–29) fraction demonstrated significant activity against SiHa and MCF7, with IC_50_ values of 0.508 ± 0.0005 µg/mL and 0.053 ± 0.0022 µg/mL, respectively. Fraction 33 (compounds **1** and **2**) exhibited activity solely against the SiHa and MCF7 lines, with IC_50_ values of 1.345 ± 0.175 µg/mL and 2.693 ± 0.054 µg/mL, respectively. An IC_50_ value represents the amount of a compound needed to inhibit biological activity by 50%. According to the National Cancer Institute (NCI) guidelines, a crude extract is considered active if it exhibits an IC_50_ value below 20 µg/mL, whereas fractions and pure compounds are deemed active with IC_50_ values below 4.0 µg/mL [14].

## 3. Discussion

### 3.1. Biological Activity

The carrageenan-induced paw edema model remains a standard assay for evaluating the anti-inflammatory activity of natural products. Carrageenan, a polysulfated polysaccharide, triggers inflammation in two distinct phases: the early phase, characterized by the release of histamine, serotonin, and kinins, and a late phase associated with prostaglandin synthesis occurring between the second and the third hours post-injection [15]. In the present study, a slight reduction in edema was observed 1 h after carrageenan administration across all treatment groups, followed by a progressive increase at subsequent time points (2, 3, and 4 h). Previous studies have reported anti-inflammatory activities of 58.3% and 50.5% for dichloromethane and methanol crude extracts obtained from wild and transformed cultures of *L. racemosa*, respectively [9,10]. In comparison, the dichloromethane extract derived from the transformed cell line LRTC3.1 demonstrated slightly increased inhibition, which may be attributed to a higher concentration of bioactive secondary metabolites exerting synergistic effects.

This study provides further confirmation that the callus generated from the LRTC3.1 transformed line maintains anti-inflammatory activity, likely due to the significant increase in IL-10 production observed compared to the vehicle control. IL-10 is well recognized for its potent anti-inflammatory properties, exerting its effects through the suppression of proinflammatory cytokines such as TNF-α, whose levels were reduced in this study. The more pronounced elevation of IL-10, relative to IL-4, may reflect its more central role in limiting inflammation and preventing tissue damage, whereas IL-4 primarily influences macrophage polarization (M1/M2 phenotypes). Moreover, IL-10 functions as a key regulatory cytokine involved in vascular homeostasis through modulation of vasodilation and vasoconstriction. Collectively, these findings suggest that IL-10 serves as the principal mediator responsible for the downregulation of TNF-α levels observed [16,17,18,19].

TNF-α has been identified as a central modulator of the inflammatory cascade, and its excessive production is associated with the pathogenesis of several autoimmune and chronic inflammatory diseases. Consequently, TNF-α inhibitors, including small molecules such as triterpenoids, have gained attention for their dual capacity to suppress proinflammatory mediators and promote anti-inflammatory cytokines [20,21]. In our study, meloxicam treatment resulted in elevated IL-4 levels without a corresponding increase in IL-10, suggesting that its anti-inflammatory action may predominantly rely on IL-4-mediated modulation of macrophage phenotype. Conversely, extracts derived from the LRTC3.1 callus line induced a robust increase in IL-10 production independently of IL-4, indicating that their anti-inflammatory effect may involve a direct stimulatory effect on IL-10 pathways. This observation supports the hypothesis that pentacyclic triterpenes present in these extracts contribute to IL-10 induction and TNF-α suppression through alternative regulatory mechanisms [10,16,18].

TNF-α is a multifunctional cytokine, reported to be present in various chronic inflammatory, autoimmune, and viral diseases, such as rheumatoid arthritis, systemic lupus erythematosus, diabetes, and SARS-CoV-2. The latter, of global significance, is characterized by an increase in proinflammatory cytokines in what is termed a “cytokine storm”, including IL-2, IL-7, interferon (IFN), and TNF-α. In this context, di- and triterpenes have been shown to interact with the SARS-CoV-2 NSP16-NSP10 enzyme. This interaction occurs in the same way as the sinefungin inhibitor, which prevents viral replication [22,23]. General terpene compounds have been studied for their positive effects in inhibiting COX-2, an enzyme responsible to produce prostaglandins, which in turn stimulate the production of proinflammatory cytokines. These compounds also exhibit a wide range of biological activities that can be utilized and are associated with a lower risk of side effects compared to many compounds found on the market such as non-steroidal drugs [24]. *L. racemosa* has been previously recognized as a rich source of terpenoid compounds with demonstrated anti-inflammatory properties in its crude extracts. While triterpenes such as maslinic acid and corosolic acid have been individually reported to exhibit significant anti-inflammatory activity [25,26], in the present study, the isolated esterified compounds—3-O-[(E)-feruloyl]-maslinic acid and 3-O-[(E)-feruloyl]-corosolic acid—did not exhibit a comparable anti-inflammatory effect. This may suggest that the addition of the feruloyl moiety could interfere with or attenuate their anti-inflammatory activity. Nevertheless, further studies are required to confirm this hypothesis. Interestingly, these esterified compounds demonstrated enhanced cytotoxic activity, indicating that the structural modification may instead potentiate their antiproliferative properties.

The anticancer activity of pentacyclic triterpenes is well established. These compounds induce apoptosis by upregulating pro-apoptotic proteins or via mitochondrial dysfunction and activation of caspases-8 and -3, as demonstrated for betulinic, oleanolic, and maslinic acids. They also inhibit cell proliferation by arresting the cell cycle through modulation of cyclin-dependent kinases (CDKs). Notably, maslinic acid further suppresses the IL-6/JAK/STAT3 signaling cascade, regulates autophagy, and inhibits angiogenesis by impairing the formation of new blood vessels that support tumor growth [27,28].

The cytotoxic activity of *L. racemosa* root extracts has been evaluated against several cancer cell lines, but only the HCT-15 data are directly comparable. Moreno-Anzures et al. [10] reported IC_50_ values of 3.14 ± 0.03 µg/mL for the crude extract and 3.32 ± 0.03 µg/mL for fraction C1F3, indicating that the crude extract is slightly more potent against HCT-15 cells. However, in this study, fraction FD (28–29) and fraction 33 containing compounds **1** and **2** demonstrated superior cytotoxicity against SiHa and MCF7 cells compared to the ExtDM.

Yu et al. [29] evaluated the anticancer activity of maslinic acid in various in vitro and in vivo models, reporting an IC_50_ of 55.20 µM (26.1 µg/mL) against the MCF7 cell line. In contrast, in our study, the feruloylated derivative of maslinic acid achieved an IC_50_ of 2.69 µg/mL, demonstrating a marked potentiation of cytotoxic activity upon conjugation with ferulic acid. Moreover, fraction FD (28–29) also exhibited strong cytotoxic activity; however, its chemical composition remains to be elucidated in follow-up investigations.

Few studies explore the biological activities of compounds **1** and **2**. Liu et al. [30] report that compound **1**, isolated from *Rhododendron latoucheae* leaves, displays potent activity against HSV-1 (herpes simplex virus-1) in Vero cells, with IC_50_ values of 2.87 μM. Another study [31] discusses the inhibitory activity of PTP1B (protein tyrosine phosphatase 1B), known for its involvement in the negative regulation of insulin signaling. It is mentioned that the pharmacological inhibition of this enzyme can contribute to the treatment of type 2 diabetes (DM2) [28].

### 3.2. Genetic Stability

The roles of *rol* genes are recognized as visual markers that can confirm genetic transformation by *A. rhizogenes*. They modify the phenotype of the plants, leading to the growth of hairy roots, which might be related to an increase in their hormone levels, particularly auxins [32]. However, it has also been suggested that these genes play a significant part in promoting the production of secondary metabolites. The *rol*B and *rol*C genes, which have been most studied, are known to be mainly involved in producing various compounds [33,34]. The *rol*B gene is the primary inducer of secondary metabolism, but it also appears to suppress cell proliferation. In contrast, the *rol*C gene not only contributes to the activation of secondary metabolism but also positively correlates with cell proliferation. Thus, there exists an antagonistic effect between these genes, with evidence even suggesting that the *rol*C gene can counteract the *rol*B gene’s effect on cell growth [16,18,35]. Regarding cell line LRTC 3.1, it is crucial to validate the genetic transformation as the transformed line of *L. racemosa* reportedly contains compounds that exhibit more activity than those in wild plants.

## 4. Materials and Methods

### 4.1. Biomass Production from Friable Callus of the LRTC3.1 Line

A friable callus culture generated from the transformed hairy root line LRT3.1 of *L. racemosa* obtained by Moreno-Anzúrez et al. [10] is renamed LRTC3.1 and used for this work. Briefly, leaves explants from *L. racemosa* seedlings germinated in vitro were infected with the *A. rhizogenes* strain ATCC15834/pTDT, which induced several hairy roots that protruded from different explants; more than 100 hairy roots were separated from the infected explants for selection, between all of these hairy roots obtained, the line LRTC3.1 was selected as a hairy root, but shortly after, it changed its growth habit spontaneously, growing as a callus and remaining to date in this form, being the plant material of this work. To produce enough plant material of this callus culture, it was cultivated in 250 mL glass flasks containing 50 mL of semi-solid medium MS/B5 composed with the micro- and macronutrients of the MS medium [36], vitamins of B5 medium [37], and 30 g/L of sucrose as a carbon source were added. It was adjusted to pH 5.7 and sterilized at 121 °C for 25 min.

### 4.2. DNA Extraction

To evaluate the genetic stability of the transformed line, PCR analysis was conducted. DNA was extracted from the cell line LRTC3.1, the *A. rhizogenes* strain ATCC15834/pTDT, and from wild-type leaves of *L. racemosa* using the plant DNAzol reagent (Invitrogen, Carlsbad, CA, USA) following the manufacturer’s protocol. For the cell lysate from the callus culture, 600 mg of 15-day-old callus was used. The sample was placed in a sterile, pre-cooled mortar. Subsequently, 1.2 mL of the plant DNAzol reagent and 10 µg/mL of RNase A (NEB, Ipswich, MA, USA) were added. The sample was ground until it formed a uniform mixture, which was then gently shaken in microcentrifuge tubes for 5 min. We then added 0.3 mL of chloroform, mixed vigorously, and centrifuged at 13,500 rpm. The aqueous phase was transferred to new tubes and the DNA was precipitated by adding 0.225 mL of 100% ethanol. Ultimately, the DNA pellet was dried and resuspended in 75 µL of TE buffer pH 8.0. For DNA isolation of *A. rhizogenes* ATCC15834/pTDT strain, one colony was inoculated in 25 mL of yeast mannitol (YM) liquid medium that contained 100 mg/L of spectinomycin and incubated in the dark at 28 ± 1 °C until it achieved an approximate optical density (OD) of 0.5 at 600 nm. Bacterial DNA extraction was carried out according to the procedure provided by the manufacturer, using the AllPrep Bacterial DNA/RNA/Protein Kit (QIAGEN, Hilden, Germany). Eventually, the samples were dried in a laminar flow chamber and resuspended in 75 µL of TE buffer with a pH of 8.0.

### 4.3. PCR Analysis

For this analysis, PCR amplifications were performed following the protocol outlined in the PCRBIO Taq Mix Red kit (PCR BIOSYSTEMS, London, UK) using the oligos reported by Bonhomme et al. [38]. For the *rol*B gene, a fragment of 652 bp was amplified using the forward sequence 5′-ACT ATA GCA AAC CCC TCC TGC-3′ and the reverse sequence 5′-TTC AGG TTT ACT GCA GCA GGC-3′. To amplify a 490 bp fragment of the *rol*C gene, we used the forward sequence 5′-TGT GAC AAG CAG CGA TGA GC-3′ and the reverse sequence 5′-GAT TGC AAA CTT GCA CTC GC-3′. In the case of the *vir*D2 gene, a 338 bp fragment was amplified using the forward oligo sequence 5′-ATG CCG ATC GAG CTC AAG T-3′ and the reverse sequence 5′-CCT GAC CCA AAC ATC TCG GCT GCC A-3′. The samples were prepared in 200 µL PCR tubes on ice to achieve a total reaction volume of 50 µL, which consisted of 0.5 µL DNA template, 25 µL of 2X PCRBIO Taq Mix, 2 µL of each primer (10 µM), and 20.5 µL of nuclease-free water. Each PCR reaction was amplified in a thermocycler (AppThermocycler SimpliAmp Thermal Life Technologies, Thermo Fisher Scientific, Bremen, Germany) under the following conditions: 1 cycle at 95 °C for 1 min, followed by 40 cycles of denaturation at 95 °C for 15 s, annealing at 55 °C for 15 s, and extension at 72 °C for 30 s. This was finalized with one cycle at 72 °C for 5 min. PCR products were analyzed by electrophoresis on a 1.5% agarose gel (Vivantis^®^) and visualized via staining with ethidium bromide.

### 4.4. Isolation of the Triterpene Ester

#### 4.4.1. General Experimental Procedures

NMR spectra were obtained on an Agilent Technologies 600 MHz DD2 spectrometer in one dimension (^1^H-NMR, ^13^C-NMR, DEPT) and two dimensions (^1^H-^1^H COSY, HSQC, and HMBC), using deuterated chloroform (CDCl_3_) as the solvent. Chemical shift data were referenced to the internal control tetramethylsilane (TMS). Coupling constants (J in Hz) and multiplicity were determined. Thin-layer chromatography was conducted using 20 × 20 cm aluminum plates thin-layer chromatography (TLC) silica gel 60F_254_ (Merck, Darmstadt, Germany). High-performance liquid chromatography analyses were completed using a Waters Alliance 2695 HPLC Separation module system, which was equipped with a photodiode array detector. Acetonitrile (CH_3_CN, HPLC grade ≥ 99.9%) and water (H_2_O, HPLC-grade) were purchased from Tecsiquim (Toluca, Mexico). Mass spectra were obtained using a Waters triple-quadrupole mass spectrometer (Wates Corporation, Milford, MA, USA) equipped with an electrospray ionization (ESI) source. The ionization source was heated to 150 °C, with a desolvation temperature of 450 °C and a nitrogen gas flow rate of 900 L/h. Argon was used as the collision gas at a flow rate of 0.10 mL/min (Thermo Fisher Scientific, Bremen, Germany), following the protocol of González-Cortazar [39].

#### 4.4.2. Obtaining the Crude Extract

Plant material (900 g callus wet weight) was dried in a 20 L Binder™ oven, 230 V 1–50/60 Hz at 30 °C for 48 h, obtaining 80.75 g dry weight. It was ground until obtaining a powder that was macerated using dichloromethane: methanol CH_2_Cl_2_:CH_3_OH (1:1; *v*/*v*) at a biomass/solvent ratio of 1:10 (*w*/*v*) for 72 h in triplicate. The extract was filtered using Whatman No. 1 filter paper and a funnel. The extract was concentrated using a rotary evaporator (Heidolph Scientific Products GmbH, Schwabach, Germany) at 40 °C to obtain 24 g of crude extract (ExtDM).

#### 4.4.3. Obtaining Compounds **1** and **2** from ExtDM

The chromatographic separation was based on the methodology described [10], where the objective of this work was to isolate the active principles, terpenoids and phytosterols, previously reported in transformed cell lines.

The extract (24 g) was subjected to column chromatography on Silica Gel 60 (30 g; Merck, Darmstadt, Germany) using a gradient system of dichloromethane and methanol (analytical grade; J.T. Baker, Phillipsburg, NJ, USA), with the polarity progressively increased in the following ratios: 100:0, 99:1, 98:2, 97:3, 96:4, 95:5, 90:10, 80:20, 70:30, 60:40, 50:50, and 0:100%. A total of 250 mL aliquots were collected and concentrated under reduced pressure using a rotary evaporator (Heidolph G3, Schwabach, Germany), yielding 50 fractions.

Subsequently, the fractions were analyzed by thin-layer chromatography (TLC) on silica gel 60 F_254_ plates (Merck, Darmstadt, Germany). Visualization was performed under ultraviolet light at 254 and 302 nm, followed by development with ammonium cerium sulfate and heating to observe the compounds. The chemical profile observed on TLC allowed grouping into four fractions: FA (12–15), FB (19–20), FC (21–23), and FD (28–29).

Chemical content observed by TLC allowed to identify that the compounds sought (terpenes and phytosterols) were present in the FA fraction (12–15, 38 mg) so it was purified by reverse phase column chromatography on silica gel RP-18 (1.0 g; Merck) using a H_2_O:CH_3_CN gradient system (analytical grade; J.T. Baker, Phillipsburg, NJ, USA) as the mobile phase, starting with 80:20 and increasing 10% of acetonitrile until culminating with a 40:60 mixture, collecting six fractions of 10 mL. In fraction 33 (12 mg), a precipitate formed and showed a single spot on TLC, which was identified as the mixture of 3-O-[(E)-feruloyl]-maslinic (**1**) and 3-O-[(E)-feruloyl]-corosolic (**2**) acids in a 60:40 ratio, respectively (Scheme 1).

### 4.5. Anti-Inflammatory Activity

#### 4.5.1. Carrageenan-Induced Inflammation Model

Female ICR (Institute of Cancer Research) mice, weighing between 30 and 40 g, were used in this study. They were kept under controlled light/dark conditions (12/12 h) at 25 °C ± 1 °C. Labdiet 5008 chow (LabDiet, Brentwood, MO, USA) and water were provided for their nourishment. All procedures were performed in accordance with the technical specifications of the Official Mexican Norm NOM-062-ZOO-1999 for producing, caring for, and using laboratory animals [40].

Eight groups of seven female ICR mice were orally treated as follows: Group 1 consisted of healthy individuals who were administered 1% Tween 20 as treatment. Group 2 involved individuals who did not receive any treatment, serving as the control group. Group 3 comprised individuals to whom meloxicam (10 mg/kg) was administered as an anti-inflammatory treatment. Group 4 consisted of individuals treated with a ExtDM (200 mg/kg). Group 5 involved individuals treated with the FB (19–20) fraction. Group 6 comprised individuals treated with the FC (21–23) fraction (20 mg/kg). Group 7 consisted of individuals treated with the FD (28–29) fraction (20 mg/kg). Lastly, Group 8 involved individuals treated with fraction 33, which included compounds **1** and **2** (20 mg/Kg).

Inflammation was induced by injecting 40 µL of a 1% λ-carrageenan solution (ChemCruz Biotechnology, Inc., Dallas, TX, USA) in distilled water into the subplantar region of the left paw for all groups, except group 1 which was injected with saline solution only. Both paws were measured using a vernier before the carrageenan injection (T0), 30 min after (T1), and subsequently 1, 2, 3, 4, and 24 h post-injection, as described by Amdekar et al. [41]. The percent inflammation was calculated using the following formula: difference between the size of edema in the treated left paw and the size of edema in the untreated right paw. Inflammation % = [∆Tcontrol − ∆Ttreatment/∆Tcontrol] × 100, where ∆T = size of the edema at 4 h − size of the edema at 0 h.

#### 4.5.2. Cytokine Evaluation

Mice were sacrificed 24 h later using sodium pentobarbital (90 mg/kg), followed by cervical dislocation. The spleen and both paws were removed and individually resuspended in phosphate-buffered saline (PBS) containing 1% phenylmethylsulfonyl fluoride (PMSF) (both from Sigma-Aldrich, St. Louis, MO, USA) at a 5:1 weight/volume ratio. Each organ was homogenized using a polytron to avoid overheating the sample; 15 s of homogenization was followed by 30 s of cooling on ice, repeated at least five times. The homogenized organs were then transferred to 1.5 mL microtubes (Eppendorf AG, Hamburg, Germany) and centrifuged for 7 min at 14,000 rpm. The supernatant was decanted into new Eppendorf microtubes and stored at −70 °C until use. Following manufacturer’s instructions, the concentration of cytokines IL-10 and IL-4 (anti-inflammatory cytokines) and TNF-α (proinflammatory cytokine) were measured using the commercial BD OptEIA mouse IL-4, IL10, and TNF-α ELISA kit (BD OptEIA, BD Biosciences, San Diego, CA, USA). The estimated concentrations were expressed in picograms/mL (pg/mL).

### 4.6. Cytotoxic Activity

Assays assessing cytotoxic activity were conducted using three human cancer cell lines: breast carcinoma (MCF7), cervical carcinoma (SiHa), and colon carcinoma (HCT-15). Cancer cells were cultured in RPMI-1640 medium (Sigma-Aldrich, St. Louis, MO, USA), supplemented with 10% fetal bovine serum (FBS; Sigma-Aldrich, St. Louis, MO, USA), and incubated at 37 °C, 5% atmospheric CO_2_, and 100% relative humidity. The cells were treated with crude extract at concentrations of 10 mg/mL, 5 mg/mL, 2 mg/mL, and 1 mg/mL Meanwhile, the fractions C2F28–29 and C3F33 (compounds **1** and **2**) were used at concentrations of 100 µg/mL, 50 µg/mL, 10 µg/mL, and 1 µg/mL. The sulforhodamine B (SRB) assay, as described by Vichai and Kirtikara [42], was employed to determine the cytotoxicity of the compounds. Cisplatin (0.5 mg/mL) served as the positive control; the negative controls comprised combined DMSO (0.1%) with Tween 80 (0.0015%) and cells cultured under identical conditions but without any treatment (control). Cells were grown to a density of 1.9 × 10^4^ per well.

### 4.7. Statistical Analysis

Data are expressed as mean ± standard error mean (SEM). Carrageenan-induced mouse paw edema and cytokine analysis were analyzed by using one-way ANOVA followed by Dunnett’s post hoc test. Differences were considered significative at *p* ≤ 0.05.

## 5. Conclusions

Interestingly, the initially expected compounds were not found in the anticipated fraction (FA12–15). Instead, a previously unreported mixture of compounds **1** and **2** was isolated and structurally characterized. To the best of our knowledge, these compounds have not been previously identified in *L. racemosa*, making this a novel contribution to the phytochemistry of the species.

This study demonstrates that the crude extract of the transformed LRTC3.1 cell line exhibits potent anti-inflammatory activity, comparable to, and in some aspects surpassing, that of commercial drugs such as meloxicam, as indicated by the reduction in TNF-α and the increase in IL-10. In terms of cytotoxicity, fractions C2F28–29 and C3F33 (compounds **1** and **2**) displayed selective activity against MCF7 breast carcinoma and SiHa cervical carcinoma cells. Furthermore, the LRTC3.1 cell line has remained stably transformed for over nine years and now exhibits a distinct phytochemical profile, notably characterized by increased levels of 3-O-[(E)-feruloyl]-maslinic acid (**1**) and 3-O-[(E)-feruloyl]-corosolic acid (**2**).

These findings highlight the biotechnological potential of LRTC3.1 as a long-term producer of bioactive metabolites. Future studies should aim to optimize the production of these compounds through the use of elicitors and to fully characterize the bioactive components in fractions C2F28–29 and other active fractions. Additionally, in vivo studies are needed to validate both the anti-inflammatory and cytotoxic effects observed in vitro, and to elucidate the underlying mechanisms of action.

## Figures and Tables

**Table 1:** Paw edema measurements in millimeters (mm) at different times in carrageenan-induced inflammation model.

Treatments	Time						
	T0 (0 min)	T1 (30 min)	T2 (1 h)	T3 (2 h)	T4 (3 h)	T5 (4 h)	T6 (24 h)
				(mm)			
Basal	1.87 ± 0.18	2.5 ± 0.48	2.19 ± 0.20	2.22 ± 0.23	2.38 ± 0.13	2.34 ± 0.22	2.02 ± 0.23
Vehicle	1.89 ± 0.11	2.38 ± 0.25	2.74 ± 0.18	2.79 ± 0.29	3.10 ± 0.12	3.38 ± 0.19	3.20 ± 0.38
Meloxicam	1.75 ± 0.07	2.35 ± 0.20	2.45 ± 0.25	2.52 ± 0.24	2.54 ± 0.16	2.71 ± 0.10	2.87 ± 0.19
* ExtMD	1.86 ± 0.11	2.48 ± 0.13	2.64 ± 0.13	2.74 ± 0.32	2.90 ± 0.32	2.68 ± 0.26	2.35 ± 0.32
FB (19–20)	1.87 ± 0.12	2.85 ± 0.37	2.48 ± 0.17	2.65 ± 0.22	2.94 ± 0.33	3.00 ± 0.33	3.15 ± 0.26
* FC (21–23)	1.80 ± 0.20	2.20 ± 0.15	2.46 ± 0.16	2.56 ± 0.13	2.65 ± 0.11	2.65 ± 0.32	2.66 ± 0.26
* FD (28–29)	1.97 ± 0.21	2.38± 0.10	2.44 ± 0.11	2.52 ± 0.09	2.74 ± 0.35	2.85 ± 0.34	2.81 ± 0.21
Mixture (**1** and **2**)	1.91 ± 0.12	2.67 ± 0.35	2.67 ± 0.33	2.75 ± 0.28	2.61 ± 0.32	2.88 ± 0.37	2.75 ± 0.41

Data expressed as the mean ± SD of 7 mice per group (* *p* ≤ 0.05 compared to the vehicle (carrageenan)).

**Table 2:** Percentage inhibition of inflammation at 4 h for the ExtDM and fractions from *L. racemosa* LRTC3.1.

Treatments	Edema (mm)	AUC (mm/min)	Inhibition of Inflammation (%)
Vehicle	3.10 ± 0.12	4448.879	–
Meloxicam	2.71 ± 0.10	3811.700	30.86
ExtDM	2.68 ± 0.26 *	3365.051	51.02
FB (19–20)	3.00 ± 0.33	4211.221	23.45
FC (21–23)	2.65 ± 0.32 *	3568.333	27.98
FD (28–29)	2.85 ± 0.34 *	3874.207	22.22
Mixture (**1** and **2**)	2.88 ± 0.37	3887.564	18.51

Data are expressed as the mean ± SD of 7 mice per group (* *p* ≤ 0.05 compared to the vehicle).

**Table 3:** Cytotoxicity (IC_50_) of ExtDM, fraction FD (28–29) and fraction 33 (compounds **1** and **2**) obtained from transformed cell line LRTC3.1 of *L. racemosa*.

Fraction/Compound	Cancer Cell Lines		
	SiHa (μg/mL)	HCT-15 (μg/mL)	MCF7 (μg/mL)
ExtDM	7.777 ± 0.534	4.837 ± 0.129	2.507 ± 0.121
FD (28–29)	0.508 ± 0.0005	5.356 ± 0.571	0.053 ± 0.0022
Mixture **1** and **2**	1.345 ± 0.175	>100	2.693 ± 0.054

IC_50_ ± SD.

**Figure A9:**
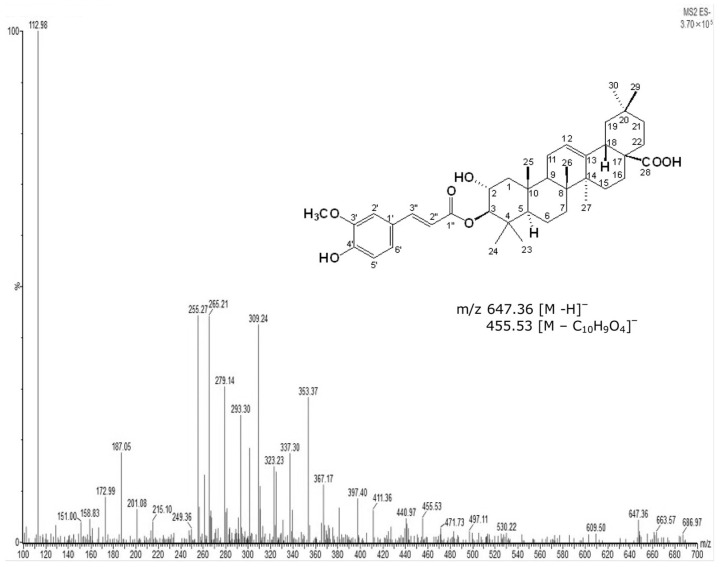
ESI mass spectrum of compound **1**.

## Data Availability

The datasets used and/or analyzed during the current study are available from the corresponding authors on reasonable request.

## Appendix A

**Table A1:** NMR spectroscopic data of compounds **1** and **2** (500 MHz, CDCl_3_, δ ppm).

Position	δ ^1^H 1	δ ^13^C 1	δ ^1^H 2	δ ^13^C 2
1	a 1.99 (m) b 1.09 (m)	48.4	a 1.99 (m) b 1.09 (m)	48.4
2	3.86 (m)	66.83	3.86 (m)	66.8
3	4.63 (d, br, 9.7 Hz)	84.94	4.63 (d, br, 9.7 Hz)	84.9
4	---	40.1	---	40.1
5	---	55.8	---	55.8
6	---	19.10	---	19.08
7	---	33.7	---	33.7
8	---	40.4	---	40.2
9	---	48.27	---	48.3
10	---	38.7	---	38.7
11	---	23.75	---	24.24
12	5.26 (m)	122.8	5.26 (m)	126.03
13	---	145.07	---	139.4
14	---	40.4	---	40.4
15	---	28.42	---	28.79
16	---	24.12	---	25.66
17	---	46.86	---	42.9
18	---	41.9	---	53.8
19	---	46.7	---	39.9
20	---	31.3	---	39.8
21	---	34.4	---	31.3
22	---	33.46	---	37.6
23	0.89 (s)	29.10	0.93 (s)	29.14
24	0.94 (s)	18.33	0.89 (s)	17.70
25	1.05 (s)	17.05	1.07 (s)	17.18
26	0.82 (s)	17.62	0.82 (s)	17.57
27	1.22 (s)	26.31	0.95 (s)	23.89
28	---	178.9	---	178.6
29	1.28 (s)	30.34	0.86 (d, 7)	21.48
30	1.22 (s)	26.31	0.97 (d, 6.8)	18.39
1′	---	127.6	---	127.6
2′	7.33 (br, s)	111.2	7.33 (br, s)	111.2
3′	---	148.7	---	148.7
4′	---	149.9	---	149.9
5′	6.87 (d, 8.1)	116.0	6.87 (d, 8.1)	116.0
6′	7.14 (d, br, 8.1)	123.8	7.14 (d, br, 8.1)	123.8
1″	---	167.8	---	167.8
2″	6.42 (d, 15.8)	116.6	6.42 (d, 15.8)	116.6
3″	7.59 (d, 15.8)	145.2	7.59 (d, 15.8)	145.2
OCH_3_	3.92 (s)	56.3	3.92 (s)	56.3
